# SAUR15 interaction with BRI1 activates plasma membrane H^+^-ATPase to promote organ development of Arabidopsis

**DOI:** 10.1093/plphys/kiac194

**Published:** 2022-05-02

**Authors:** Mengzhan Li, Chunli Liu, Shelley R Hepworth, Chaofan Ma, Hong Li, Jia Li, Suo-Min Wang, Hongju Yin

**Affiliations:** State Key Laboratory of Grassland Agro-ecosystems; Key Laboratory of Grassland Livestock Industry Innovation, Ministry of Agriculture and Rural Affairs; College of Pastoral Agriculture Science and Technology, Lanzhou University, Lanzhou 730000, People’s Republic of China; State Key Laboratory of Grassland Agro-ecosystems; Key Laboratory of Grassland Livestock Industry Innovation, Ministry of Agriculture and Rural Affairs; College of Pastoral Agriculture Science and Technology, Lanzhou University, Lanzhou 730000, People’s Republic of China; State Key Laboratory of Grassland Agro-ecosystems; Key Laboratory of Grassland Livestock Industry Innovation, Ministry of Agriculture and Rural Affairs; College of Pastoral Agriculture Science and Technology, Lanzhou University, Lanzhou 730000, People’s Republic of China; Department of Biology, Institute of Biochemistry, Carleton University, Ottawa, Ontario K1S 5B6, Canada; State Key Laboratory of Grassland Agro-ecosystems; Key Laboratory of Grassland Livestock Industry Innovation, Ministry of Agriculture and Rural Affairs; College of Pastoral Agriculture Science and Technology, Lanzhou University, Lanzhou 730000, People’s Republic of China; State Key Laboratory of Grassland Agro-ecosystems; Key Laboratory of Grassland Livestock Industry Innovation, Ministry of Agriculture and Rural Affairs; College of Pastoral Agriculture Science and Technology, Lanzhou University, Lanzhou 730000, People’s Republic of China; Ministry of Education Key Laboratory of Cell Activities and Stress Adaptations, School of Life Sciences, Lanzhou University, Lanzhou 730000, People’s Republic of China; School of Life Sciences, Guangzhou University, Guangzhou 510006, People’s Republic of China; State Key Laboratory of Grassland Agro-ecosystems; Key Laboratory of Grassland Livestock Industry Innovation, Ministry of Agriculture and Rural Affairs; College of Pastoral Agriculture Science and Technology, Lanzhou University, Lanzhou 730000, People’s Republic of China; State Key Laboratory of Grassland Agro-ecosystems; Key Laboratory of Grassland Livestock Industry Innovation, Ministry of Agriculture and Rural Affairs; College of Pastoral Agriculture Science and Technology, Lanzhou University, Lanzhou 730000, People’s Republic of China

## Abstract

Brassinosteroids (BRs) are an important group of plant steroid hormones that regulate growth and development. Several members of the SMALL AUXIN UP RNA (SAUR) family have roles in BR-regulated hypocotyl elongation and root growth. However, the mechanisms are unclear. Here, we show in Arabidopsis (*Arabidopsis thaliana*) that SAUR15 interacts with cell surface receptor-like kinase BRASSINOSTEROID-INSENSITIVE 1 (BRI1) in BR-treated plants, resulting in enhanced BRI1 phosphorylation status and recruitment of the co-receptor BRI1-ASSOCIATED RECEPTOR KINASE 1. Genetic and phenotypic assays indicated that the SAUR15 effect on BRI1 can be uncoupled from BRASSINOSTEROID INSENSITIVE 2 activity. Instead, we show that SAUR15 promotes BRI1 direct activation of plasma membrane H^+^-ATPase (PM H^+^-ATPase) via phosphorylation. Consequently, SAUR15–BRI1–PM H^+^-ATPase acts as a direct, PM-based mode of BR signaling that drives cell expansion to promote the growth and development of various organs. These data define an alternate mode of BR signaling in plants.

## Teaser

SAUR15–BRI1-plasma membrane (PM) H+-ATPase acts as a direct, PM-based mode of brassinosteroid signaling that drives cell expansion to promote plant growth and development.

## Introduction

Brassinosteroids (BRs) are a class of polyhydroxylated steroid hormones, first discovered in pollen extracts and later found in all growing tissues of vascular plants (Grove et al., 1979; Kauschmann et al., 1996; Fujioka and Sakurai, 1997). Studies of mutants with defects in BR biosynthesis or signaling indicated that BRs are essential for nearly all aspects of plant growth and development. Mutants exhibit a range of developmental defects, such as extreme dwarfism, reduced hypocotyl and primary root elongation, and impaired lateral root formation (Clouse and Sasse, 1998; Gudesblat and Russinova, 2011; Wang et al., 2012; Kim and Russinova, 2020; Lv and Li, 2020). Substantial progress has been made in uncovering the molecular mechanisms of BR signaling and BR-regulated gene expression.

BRs are perceived at the plasma membrane (PM) by the extracellular domain (ED) of BRASSINOSTEROID-INSENSITIVE 1 (BRI1), a leucine-rich repeat receptor-like kinase (LRR-RLK) protein (Li and Chory, 1997), and its co-receptor, BRI1-ASSOCIATED RECEPTOR KINASE 1 (BAK1, also known as SOMATIC EMBRYOGENESIS RECEPTOR KINASE 3), another LRR-RLK protein, which is involved in many signaling processes (Li and Chory, 1997; Li et al., 2002; Nam and Li, 2002; Gou et al., 2012). BRI1 and BAK1 sequentially phosphorylate each other in their cytosolic kinase domains via autophosphorylation and transphosphorylation to activate signaling (Wang et al., 2008, 2012). This signaling leads to inhibition of glycogen synthase kinase 3-like kinase BRASSINOSTEROID-INSENSITIVE 2 (BIN2; Li et al., 2001; Li and Nam, 2002; Gampala et al., 2007; Peng et al., 2008) and subsequent nuclear accumulation of nonphosphorylated forms of the transcription factors BRASSINAZOLE-RESISTANT 1 (BZR1) and BRI1-EMS-SUPPRESSOR 1 (BES1; Wang et al., 2002; Yin et al., 2002; Zhao et al., 2002). Activated BZR1/BES1 interact with other transcriptional regulators in the nucleus to regulate the expression of thousands of BR responsive genes, including genes involved in BR biosynthesis, such as *CONSTITUTIVE PHOTOMORPHOGENIC DWARF* (*CPD*), *DWARF4* (*DWF4*), and *ROTUNDIFOLIA3* (Mathur et al., 1998; Noguchi et al., 2000; Kim et al., 2005), and various members of the *SMALL AUXIN UP RNA* (*SAUR*) gene family (Yin et al., 2005; Sun et al., 2010; Yu et al., 2011; Walcher and Nemhauser, 2012; Oh et al., 2014; Ren and Gray, 2015).

Recent studies implicate SAURs in diverse developmental processes. For example, SAUR36, SAUR41, SAUR63, and SAUR19 subfamily members promote cell expansion and elongation (Chae et al., 2012; Spartz et al., 2012; Kong et al., 2013; Stamm and Kumar, 2013). Sun et al. (2016) suggested that SAUR50 and SAUR65 aid in cotyledon expansion and hypocotyl elongation. Similarly, our previous study showed that overexpression of *SAUR15* confers an increase in hypocotyl length and the number of lateral roots (Yin et al., 2020). Mechanistically, these SAURs were shown to inhibit D-clade type 2C protein phosphatases (PP2C-Ds) to activate PM H^+^-ATPases that promote cell expansion (Spartz et al., 2014; Ren et al., 2018; Wong et al., 2019; Copeland, 2020; Yin et al., 2020). Several of these *SAURs* are induced by BR signaling and participate in BR-regulated processes (Yin et al., 2005; Sun et al., 2010; Yu et al., 2011; Walcher and Nemhauser, 2012; Oh et al., 2014; Ren and Gray, 2015; Minami et al., 2019), but how SAUR activity is integrated with BR signaling machinery is unclear. It has been suggested that BR initiates cell enlargement by activating PM H^+^-ATPases (Caesar et al., 2011; Minami et al., 2019). Caesar et al. (2011) indicated that BRI1 could interact with and activate PM H^+^-ATPases, but the evidence for how BRI1 promotes the activity of PM H^+^-ATPase is insufficient. In addition, Minami et al. (2019) demonstrated that BR elevates PM H^+^-ATPases activity via transcriptional regulation of *SAURs* under the traditional BRI1–BIN2 pathway, which subsequently activate PM H^+^-ATPases by inhibiting PP2C-Ds. Since PM H^+^-ATPases could be phosphorylated and activated through TMK-based auxin signaling (Li et al., 2021; Lin et al., 2021), kinases responsible for phosphorylation of PM H^+^-ATPases in response to BR and the associated mechanism remain to be clarified.

We show here that BR stimulates SAUR15 interaction with BRI1 receptor leading to the phosphorylation and activation of PM H^+^-ATPase. This mode of direct signaling connects PM-localized SAUR activity to BR-regulated cell expansion important for leaf enlargement, hypocotyl elongation, and lateral root formation.

## Results

### *SAUR15* mutants show reduced sensitivity to BR whereas *SAUR15*-OE lines have increased sensitivity to BR

Our previous study showed that SAUR15 promotes lateral root initiation by inhibiting PP2C-Ds to activate PM H^+^-ATPases involved in cell expansion (Yin et al., 2020). The auxin-upregulated S*AUR15* gene is also marker for BR-induced gene expression (Yin et al., 2005). As shown in Supplemental Figure S1, when treated with 2,4-epibrassinolide (BL, a bioactive BR), *SAUR15* transcripts were 3.5-fold induced in wild-type (Columbia-0, Col-0) and further increased in *BRI1*-OE plants (*35S::BRI1-FLAG*). Lower than wild-type induction was observed in BR signaling-impaired mutants *bri1-301*, a weak allele of *BRI1* (Xu et al., 2008), and in *bin2-1* (+/−), a *BIN2* gain-of-function mutant (Li and Nam, 2002) (Supplemental Figure S1, A–C). There were no substantial changes relative to wild-type seedlings observed in *bri1-701*, a null allele of *BRI1* (Gou et al., 2012) (Supplemental Figure S1A), and *bin2-1* (−/−) (Li and Nam, 2002) (Supplemental Figure S1A) treated with or without BL (Supplemental Figure S1, B and C). Transcripts of *SAUR15* were substantially lower in BR biosynthesis mutants *cpd91*, a weak allele of *CPD* (Du et al., 2017), and *cpd*, a null allele of *CPD* (Du et al., 2017) without BL but rose to similar levels as Col-0 with BL treatment (Supplemental Figure S1, A and B). These data show that *SAUR15* expression is sensitive to BR and prompted us to speculate if SAUR15 functions in the BR signaling pathway.

To test this hypothesis, we measured the root inhibition response of *saur15-1* mutant and *SAUR15*-OE lines (*35S::SAUR15-FLAG* and *pSAUR15::SAUR15-FLAG*) compared to wild-type seedlings treated with BL. Our previous study showed that *saur15-1* mutants have smaller cotyledons, shorter hypocotyls, and fewer secondary (i.e. lateral and adventitious) roots whereas *SAUR15*-OE lines have the reverse phenotype (Yin et al., 2020). Root measurements showed that *saur15-1* mutants were less sensitive to BL, whereas S*AUR15*-OE lines were more sensitive compared to the wild type (Supplemental Figure S1, D–G). Hypocotyl elongation in the dark-grown etiolated seedlings treated with brassinazole (BRZ, a specific inhibitor of BR biosynthesis) was also assessed. Etiolated *saur15-1* seedlings showed increased sensitivity to BRZ, whereas *SAUR15*-OE lines were less sensitive compared to the wild type (Supplemental Figure S1, H–K). These data suggest that SAUR15 is a positive regulator of BR signaling.

### *SAUR15*-OE partially rescues BR biosynthesis and signaling mutant defects

**Figure 1:**
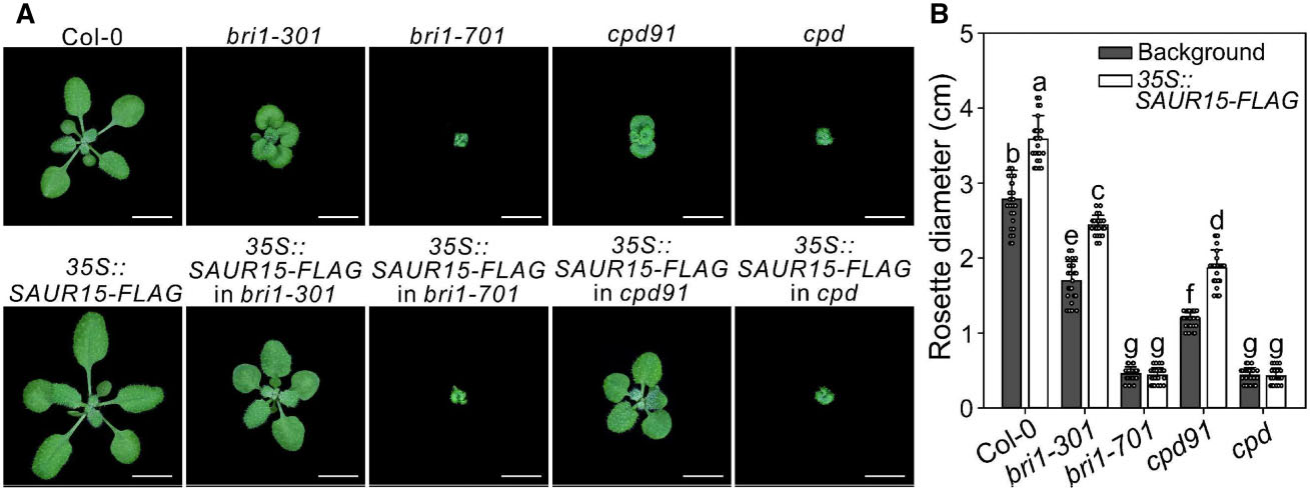
SAUR15 plays vital roles in BR-related development of Arabidopsis. A, 3-week-old wild type (Col-0), *bri1* mutants (*bri1-301* and *bri1-701*), *cpd* mutants (*cpd91* and *cpd*), and *SAUR15*-OE (*35S::SAUR15-FLAG*) seedlings grown in soil. Scale bars, 1 cm. B, Rosette diameter of seedlings in (A). Data shown in (B) are mean ± standard deviation (*n* = 25). Different letters indicate significant differences (one-way ANOVA with Tukey’s test, *P* < 0.05).

To further examine SAUR15 function, we firstly analyzed the β-galactosidase (GUS) staining pattern of *pSAUR15::GUS* transgenic plants (Yin et al., 2020). As expected, staining was observed throughout the plant (Yin et al., 2020) (Supplemental Figure S2A), suggesting that SAUR15 is a general regulator of growth. Next, *SAUR15* was overexpressed in BR signaling and biosynthesis mutants to test for complementation of growth defects (Supplemental Figure S2, B and C). Phenotyping showed that *SAUR15*-OE partially suppressed the growth defects of weak BR receptor and biosynthesis mutants *bri1-301* and *cpd91*, respectively (Figure 1; Supplemental Figure S2, D–S). For example, rosette diameter (Figure 1), root length (Supplemental Figure S2, D, E, H, and I), lateral and adventitious root numbers (Supplemental Figure S2, D, F, H, J, N, O, R, and S), and hypocotyl length (Supplemental Figure S2, D, G, H, K, L, M, P, and Q) were partially corrected. However, *SAUR15*-OE failed to complement the phenotypic defects of null mutants *bri1-701* and *cpd* (Figure 1; Supplemental Figure S2, D–S). Thus, SAUR15 regulation of BR-mediated processes is BR activated and BRI1 dependent.

### SAUR15 interacts with BRI1 in vitro and in vivo

**Figure 2:**
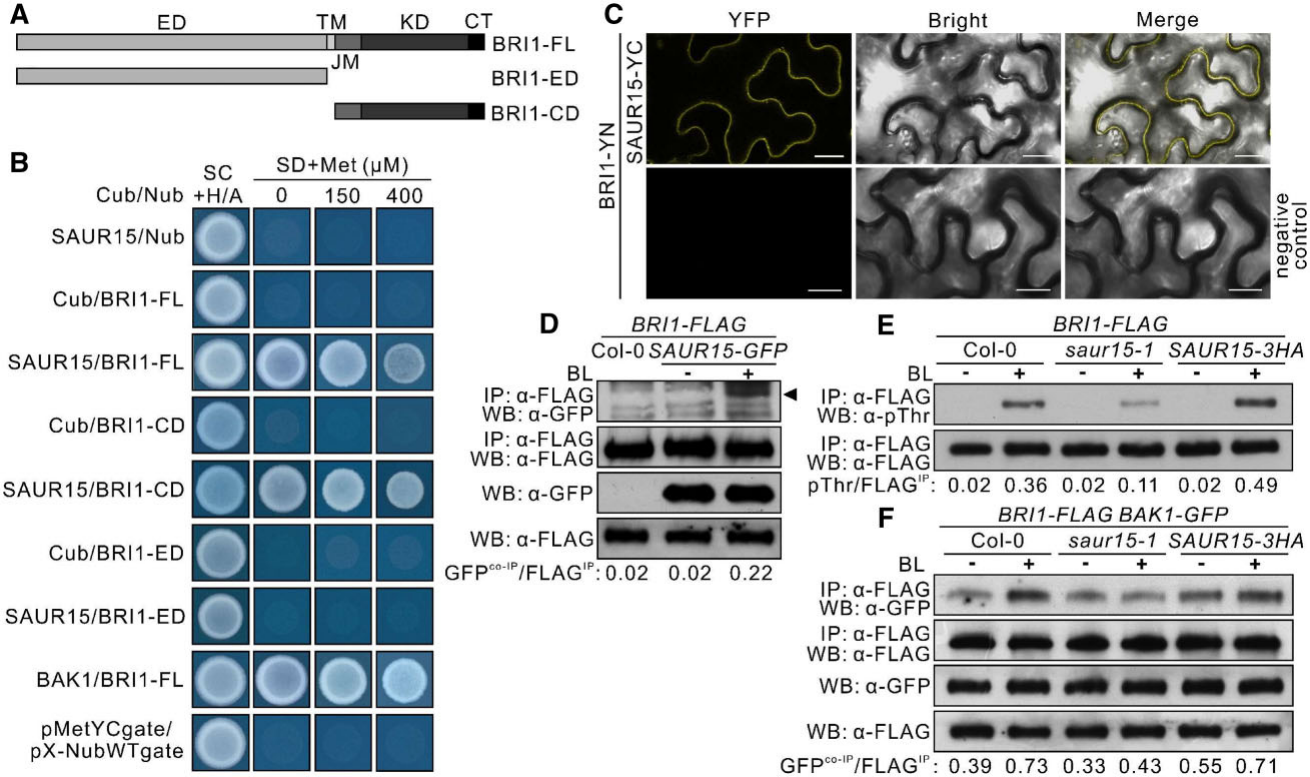
SAUR15 interacts with BRI1 and positively regulates its activity. A, Diagram of FL and truncated BRI1 constructs. TM, transmembrane domain; JM, intracellular-juxtamembrane domain; KD, kinase catalytic domain; CT, C-terminus; BRI1-FL, FL BRI1. B, Interaction assay for SAUR15 with FL and truncated BRI1 using a mating-based split ubiquitin system (mbSUS) yeast two-hybrid system. SAUR15 and BAK1 proteins were each fused to the C-terminal part of ubiquitin (Cub). BRI1-FL, BRI1-ED, and BRI1-CD proteins were each fused to the N-terminal part of ubiquitin (Nub). Yeast was grown on a SC medium containing Adenine and Histidine (SC + Ade + His) for selection of diploid cells or on a synthetic dextrose minimal medium (SD) with 150 and 400 mM or without Met for interaction detection. BRI1-FL-Nub with BAK1-Cub was used as a positive control. Empty vector pair *pMetYCgate* with *pX-NubWTgate* was used as a negative control. C, SAUR15 interacts with BRI1 in *N. benthamiana* leaf epidermal cells. SAUR15 was fused to the C-terminal part of YFP (YC); BRI1 was fused to the N-terminal part of YFP (YN). *GV3101* harboring BRI1-YN was used as the negative control. Scale bars, 10 μm. D, SAUR15 interacts with BRI1 in a BR-dependent manner. Ten-day-old seedlings grown on 1/2 MS medium were collected and treated with or without 1 μM BL for 2 h. The specific band for SAUR15-GFP was marked by an arrow. E, Phosphorylation of Threonine (Thr) residues of BRI1-FLAG in vivo. Ten-day-old liquid-cultured seedlings were collected and treated with or without 100 nM BL for 90 min. F, SAUR15 regulates the BR-induced interaction of BRI1 and BAK1. Flag-tagged BRI1 and GFP-tagged BAK1 double transgenic seedlings of Col-0, *saur15-1*, and *SAUR15-3HA* (*35S::SAUR15-3HA*) were grown in liquid medium for 10 days and then treated with or without 1 μM BL for 2 h. For (D–F), IP, immunoprecipitation; WB, western blot. The GFP^co-IP^/FLAG^IP^ ratio in (D) and (F), pThr/FLAG^IP^ ratio in (E) was measured by western blot analysis using ImageJ.

Given that SAUR15 protein is PM localized (Yin et al., 2020), we next tested if SAUR15 interacts with the BR receptor to regulate its activity. Yeast two-hybrid analyses using a mating-based split ubiquitin system (mbSUS) showed that SAUR15 can interact with the cytoplasmic domain (CD) of BRI1 protein in yeast cells (Figure 2, A and B). Bimolecular fluorescence complementation (BiFC) assays demonstrated that this interaction occurs at the PM in leaf epidermal cells of *Nicotiana benthamiana* (Figure 2C). However, co-immunoprecipitation (co-IP) assays using GFP-fused SAUR15 and FLAG-tagged BRI1 double-transgenic seedlings found little or no interaction of SAUR15 with BRI1 protein in the absence of BL (Figure 2D). Treatment with BL resulted in a clear increase of SAUR15 co-immunoprecipitated BRI1 protein (Figure 2D). These results show that SAUR15 interacts with BRI1 in vitro and in vivo, in a BR-dependent manner.

### SAUR15 positively regulates BRI1 kinase activity

To detect how these interactions between SAUR15 and BRI1 exert their functions, we tested the possibility that SAUR15 interaction regulates BRI1 activity. Immunoblotting was used to examine the phosphorylation state of BRI1 in *saur15-1* and *SAUR15-3HA* (another *CaMV35S* promoter-driven *SAUR15* overexpression line, *35S::SAUR15-3HA*) seedlings compared to wild-type seedlings treated with BL (Figure 2E). As reported previously (Wang et al., 2008), phosphorylated BRI1 was substantially enriched in wild-type seedlings upon BL treatment (Figure 2E). Immunoblotting showed that phosphorylated BRI1 was lower in *saur15-1* and higher in *SAUR15-3HA* compared to wild-type seedlings treated with BL (Figure 2E). Therefore, SAUR15 promotes phosphorylation of BRI1.

We next tested if SAUR15 promotes the association of BRI1 with BAK1. As expected, co-IP showed that BRI1 and BAK1 interaction is enhanced by treatment with BL (Figure 2F). This response was not observed in *saur15-1* seedlings where less complex formation was detected following treatment with BL (Figure 2F). Notably, the interaction between BRI1 and BAK1 was substantially enhanced in *SAUR15-3HA* plants compared to the wild type in the absence of BL. The interaction was further enhanced upon BL treatment (Figure 2F). Thus, SAUR15 promotes BRI1–BAK1 receptor complex formation.

### SAUR15 mediates a separate pathway from BIN2

Canonical BRI1–BAK1 signaling inhibits BIN2 kinase leading to the dephosphorylation of BZR1/BES1 and the downstream transcriptional responses, including the repression of BR biosynthesis genes *CPD* and *DWF4* (Mathur et al., 1998; Noguchi et al., 2000; Zhao et al., 2002; Kim et al., 2005). Phosphorylation status of BZR1 was investigated with a specific α-BZR1 antibody and transcripts for *CPD* and *DWF4* were measured in *saur15-1* mutants and *SAUR15*-OE seedlings compared to wild type treated with or without 1 μM BL for 120 min. In Col-0, two BZR1 bands with almost equal signal intensity were observed without BL treatment, while unphosphorylated BZR1 was increased dramatically and phosphorylated BZR1 disappeared upon BL treatment (Supplemental Figure S3A). In *bri1-301*, the amount of phosphorylated BZR1 was more than that of unphosphorylated BZR1 without BL treatment, and the phosphorylated BZR1 band did not disappear under BL treatment (Supplemental Figure S3A). However, the phosphorylation status changes of BZR1 in *saur15-1* and *SAUR15*-OE lines in response to BL are similar to that of Col-0 (Supplemental Figure S3A). In addition, *CPD* and *DWF4* transcripts were substantially downregulated in Col-0, by about 81% and 96%, respectively, but decreased in *bri1-301* plants by about 57% and 86%, respectively, upon BL treatment (Supplemental Figure S3, B and C). Surprisingly, the expression of *CPD* and *DWF4* in *saur15-1* decreased by about 81% and 95% upon BL treatment. In *SAUR15*-OE lines, the expression of *CPD* decreased by about 85% and 83%, and the expression of *DWF4* decreased by about 98% and 97% under BL treatment (Supplemental Figure S3, B and C). These results showed that phosphorylation of BZR1 and transcription of these BR biosynthesis genes in response to BR were not changed in *saur15-1* and *SAUR15*-OE lines compared to Col-0, suggesting that the traditional BIN2-mediated BR signaling pathway is not affected by SAUR15. Thus, SAUR15–BRI1 may not require the BIN2 activity exert its function at the PM.

**Figure 3:**
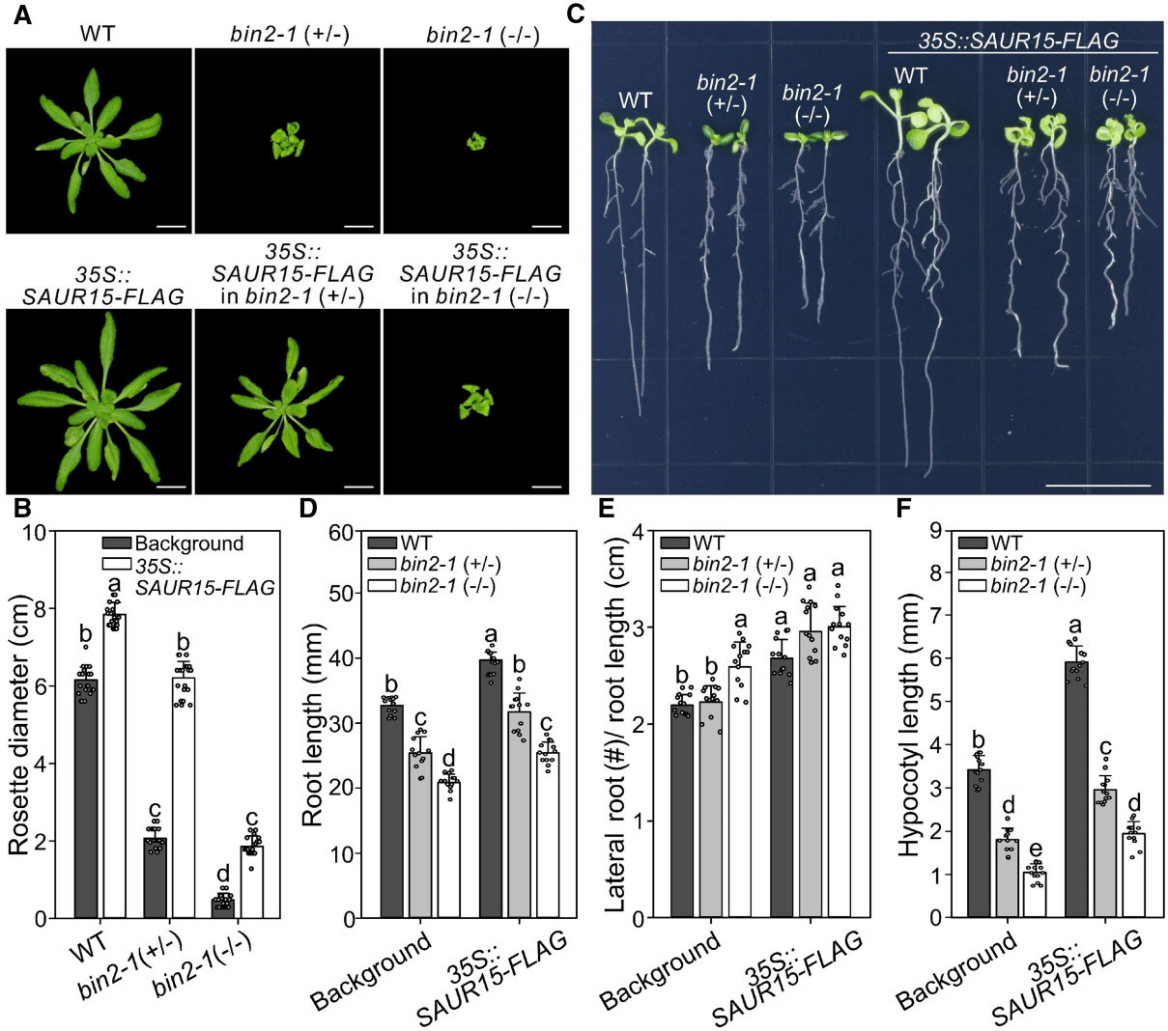
*SAUR15*-OE rescues *bin2-1*, a gain-of-function allele of *BIN2*. A, 4-week-old seedlings of WT, *BIN2* heterozygous mutant *bin2-1* (+/−), *BIN2* homozygous mutant *bin2-1* (−/−) and *SAUR15*-OE lines (*35S::SAUR15-FLAG*) grown in soil. Scale bars, 1 cm. B, Rosette diameter of seedlings in (A). C, Phenotypes of 10-day-old seedlings grown in light. Scale bars, 1 cm. D–F, Primary root length (D), lateral root density (E), and hypocotyl length (F) analysis of seedlings in (C). Data shown in (B) are mean ± standard deviation (*n* = 20). Data shown in (D–F) are mean ± standard deviation (*n* = 13). Different letters indicate significant differences (one-way ANOVA with Tukey’s test, *P* < 0.05).

To check this hypothesis, we tested if *SAUR15*-OE compensates for growth defects in *bin2-1* mutants (Supplemental Figure S3D). Indeed, *SAUR15*-OE substantially rescued *bin2-1* (+/−) defects in rosette diameter (Figure 3, A and B), root length (Figure 3, C and D), secondary root number (Figure 3, C and E; Supplemental Figure S3, G and H), and hypocotyl length (Figure 3, C and F; Supplemental Figure S3, E and F). Surprisingly, root and shoot defects were also somewhat rescued in the BR signaling-blocked *bin2-1* (−/−) mutants (Figure 3; Supplemental Figure S3, D–H). These results suggest that SAUR15–BRI1 and BRI1–BIN2 have separated functions in the BR signaling pathway and that SAUR15–BRI1 activity is retained in the *BIN2* gain-of-function mutant.

### SAURs are likely limiting for BRI1 activity in shoots

**Figure 4:**
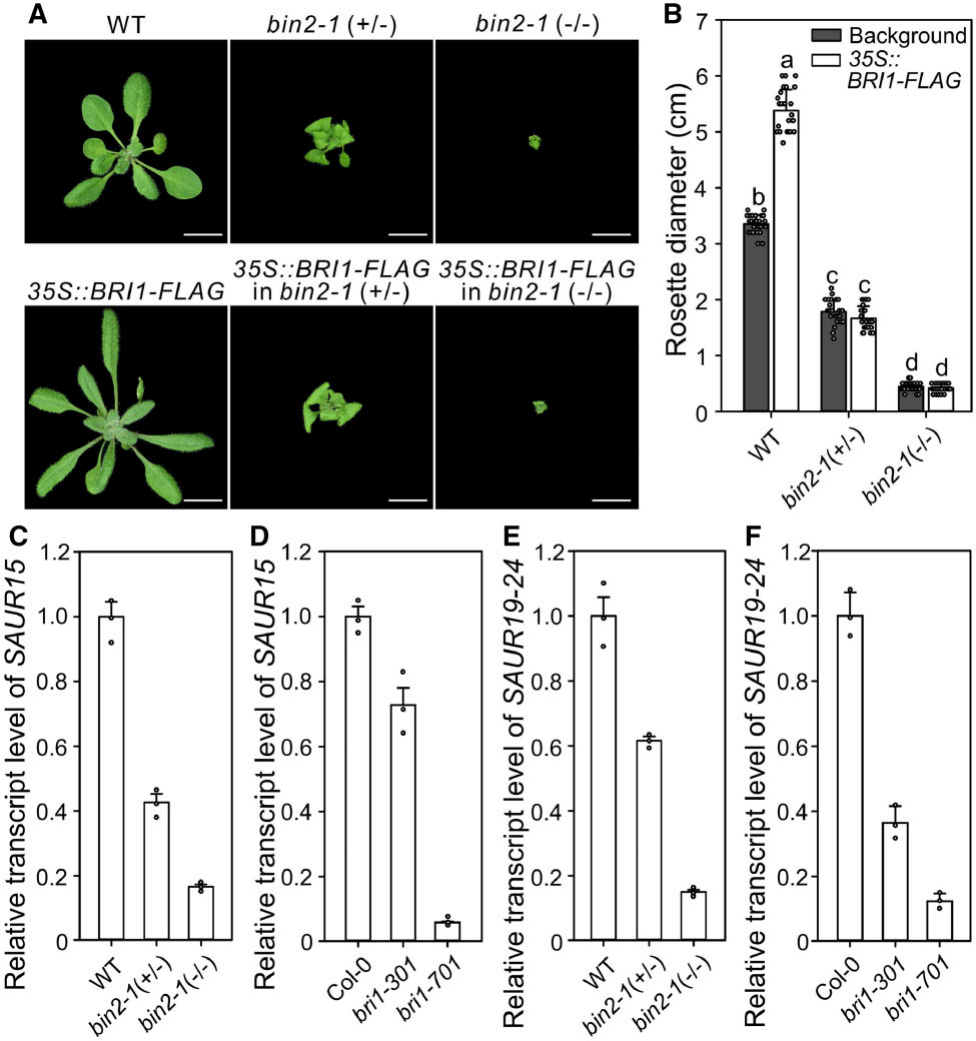
SAUR15 plays a key role in SAUR–BRI1 signaling pathway. A, *BRI1* overexpression cannot rescue *bin2-1* (+/−) or *bin2-1* (−/−)*.* Three-week-old seedlings grown in soil. Scale bars, 1 cm. B, Rosette diameter of seedlings in (A). C and D, Relative transcript level of *SAUR15* in shoots of *bin2* (C) and *bri1* (D) related seedlings grown in soil for 2 weeks. E and F, Relative transcript level of *SAUR19-24* in shoots of *bin2* (E) and *bri1* (F) related seedlings grown in soil for 2 weeks. For (B), data are mean ± standard deviation (*n* = 25). For (C–F), *ACT2* was used as the reference gene. Data show mean ± standard deviation of three technical replicates. Experiments were repeated 3 times with similar results. Each biological replicates includes shoots from one seedling in same development stage. Different letters indicate significant differences (one-way ANOVA with Tukey’s test, *P* < 0.05). Primers used in (C–F) are listed in Supplemental Table S1.

Root defects in *bin2-1* (−/−) are relatively mild compared to *bri1-701* or *cpd* mutants (Figure 3, C–E; Supplemental Figure S2, D–F and H–J), but the rosette diameter is equally impaired in these mutants (Supplemental Figure S4, A–C), suggesting that BR synthesis or BRI1 and/or SAUR15 activities are differentially expressed in the shoot. Previous studies demonstrated that the BR biosynthesis is suppressed by the feedback inhibition from BRI1–BIN2 signaling pathway (Noguchi et al., 1999; Wang et al., 2002; Yu et al., 2011), which indicated that, unlike *cpd*, BR content in *bin2-1* (−/−) and *bri1-701* is sufficient and the phenotypic defects of them is not caused by BR level. Therefore, we analyzed *BRI1* transcript accumulation in shoots of WT, *bin2-1* (+/−), and *bin2-1* (−/−) seedlings but no significant differences were observed (Supplemental Figure S4D). Meanwhile, *BRI1-*OE did not rescue the shoot phenotype of *bin2-1* (+/−) and *bin2-1* (−/−) (Figure 4, A and B; Supplemental Figure S4E), suggesting that *BRI1* transcript is not a limiting factor in these mutants. We then checked the expression of *SAUR15* and related *SAUR19* subfamily genes (*SAUR19-24*; Yin et al., 2020), which possess similar functions in cell expansion regulated growth (Spartz et al., 2012, 2014), in *bin2* and *bri1* mutant seedlings. The expression levels of these genes in *bin2-1* (+/−) and *bin2-1* (−/−) seedling shoots were much less than that in WT shoots (Figure 4, C and E; Supplemental Figure S4, F and H), and that in *bri1* mutant shoots was substantially lower than in wild-type shoots (Figure 4, D and F), with *bin2-1* (−/−) and *bri1-701* shoots showing the least expression (Figure 4, C–F; Supplemental Figure S4, F and H). On the contrary, the expression level of *SAUR15* in *bin2-1* (−/−) roots was much higher than that in WT (Supplemental Figure S4G). Comparing to WT, the expression of *SAUR19-24* in *bin2-1* (−/−) roots was also promoted (Supplemental Figure S4I), even though the transcript accumulation of this subfamily in roots is very low (Spartz et al., 2012). These combined data indicate that the phenotype of *bin2-1* mutants is highly sensitive to *SAUR15* transcript level, as further confirmation that SAUR15 plays a key role in the SAUR15–BRI1-mediated BR signaling.

### SAUR15–BRI1 positively regulates the activity of H^+^-ATPases

**Figure 5:**
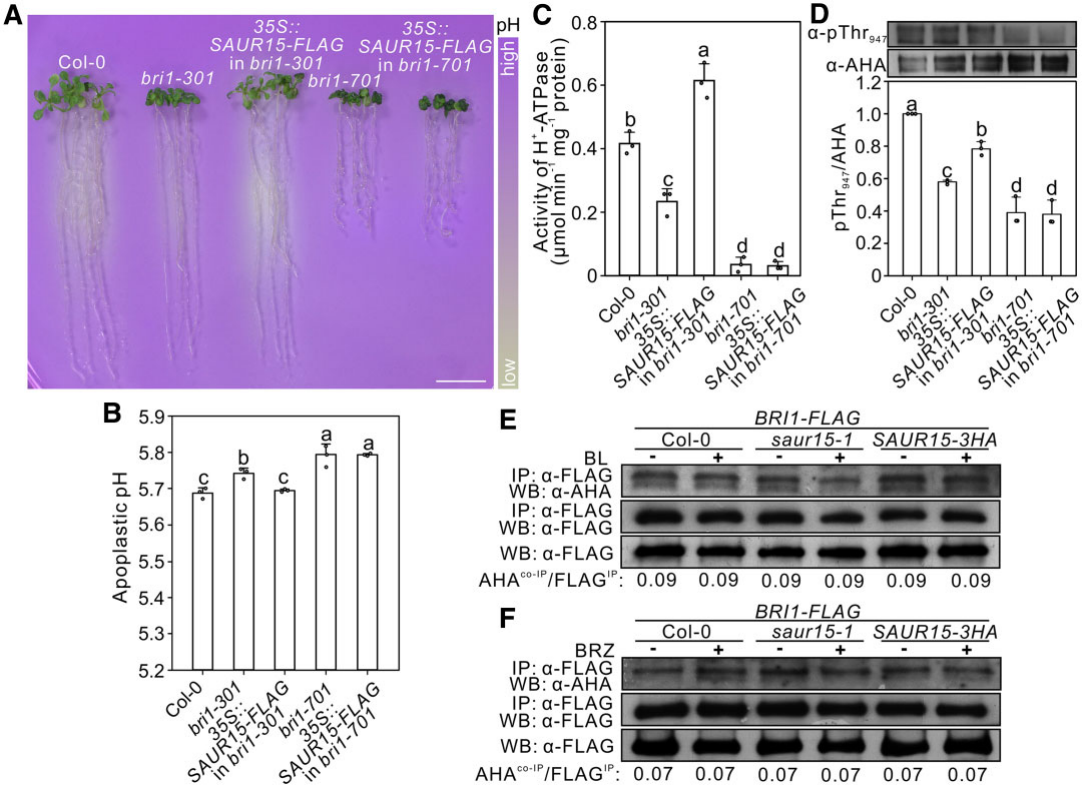
SAUR15–BRI1 positively regulates PM H^+^-ATPase activities. A, Medium acidification assays. Twelve-day-old seedlings were transferred to plates containing the pH indicator dye bromocresol purple. Color changes were recorded after 24 h. Scale bar, 1 cm. B, Leaf apoplastic pH. Absorbance of 8-hydroxypyrene-1,3,6-trisulfonic acid trisodium was measured at 510 and 530 nm. At least 50 leaves were used for each replicate. C, Relative vanadate-sensitive ATP hydrolytic activity and phosphorylation status of PM H^+^-ATPase in PM fractions with equal PM protein level. PM fractions were prepared from 7-day-old seedlings grown on 1/2 MS medium. D, Interaction between BRI1 and AHA in wild-type and *SAUR15*-related seedlings with or without (BL treatment. Seedlings expressing *BRI1-FLAG* in Col-0, *saur15-1*, and *SAUR15-3HA* (*35S::SAUR15-3HA*) genetic backgrounds were grown in 1/2 MS liquid medium for 10 days and then treated with or without 1 μM BL for 2 h. Total membrane protein was immunoprecipitated and subjected to immunoblot analysis as indicated. E, Interaction between BRI1 and AHA in wild-type and SAUR15-related seedlings treated with or without BRZ. Seedlings described in (D) were grown in 1/2 MS liquid medium for 9 days and then treated with 1 μM BRZ for 18 h. Total membrane protein was immunoprecipitated and subjected to immunoblot analysis as indicated. F, Phosphorylation state analysis for penultimate residue of AHA. The amount of PM H^+^-ATPase (AHA) and the phosphorylation state of the penultimate residue of AHA were determined with α-AHA and α-pThr_947_ separately. The value of pThr_947_/AHA in Col-0 was set to 1.00 and values of other seedlings were normalized. Average values of the three replicated results from Figure 5F and Supplemental Figure S5, H and I were shown. For (B, C, and F), data show mean ± standard deviation of three technical replicates, and different letters indicate significant differences (one-way ANOVA with Tukey’s test, *P* < 0.05). For (D and E), IP, immunoprecipitation; WB, western blot. For (D–F), the AHA^co-IP^/FLAG^IP^ ratio in (D and E) and pThr_947_/AHA ratio in (F) was measured by western blot analysis using ImageJ.

It has been suggested that a fast BR-regulated signal response links BRI1 with the phosphorylation and activation of PM H^+^-ATPases for cell expansion (Caesar et al., 2011). Indeed, SAUR proteins bind and inhibit PP2C-Ds, thereby activating PM H^+^-ATPases to promote cell expansion (Spartz et al., 2014; Minami et al., 2019). We, therefore, tested if PM H^+^-ATPases are the substrates of SAUR15–BRI1 activation. First, we tested the effect of SAUR15–BRI1 on H^+^-ATPase activity using physiological and biochemical assays. Growth on pH indicator plates showed that *bri1-301* and *bri1-701* mutants have reduced media acidification and that *BRI1-*OE seedlings have increased media acidification compared to wild type (Figure 5A; Supplemental Figure S5A). Further, *SAUR15*-OE increased the media acidification of *bri1-301* but not of *bri1-701* mutants (Figure 5A). We then assayed the apoplastic pH of rosette leaves. Consistent with the media acidification results, apoplastic pH was significantly increased in *bri1-301* and *bri1-701* but decreased in *BRI1*-OE seedlings compared to wild type (Figure 5B; Supplemental Figure S5B). *SAUR15*-OE decreased apoplastic pH in *bri1-301* but not in *bri1-701* mutants (Figure 5B). To directly assess the effect of SAUR15 and BRI1 on PM H^+^-ATPase, we measured the vanadate-sensitive ATP hydrolytic activity in PM fractions (Figure 5C). Using the same amount of PM protein as for Col-0, the ATPase activity of *bri1-301* and *bri1-701* mutants was reduced ∼44% and 92%, respectively (Figure 5C). Further, *SAUR15*-OE increased PM H^+^-ATPase activity in *bri1-301* but not in *bri1-701* mutants (Figure 5C). These data strongly suggest that BRI1 is a positive regulator of PM H^+^-ATPases and SAUR15 promotes this response via BRI1. Additionally, the medium acidification assay for *BIN2* and *SAUR15* related seedlings indicates that the PM H^+^-ATPase activity in roots of *bin2-1* seedlings is higher than those of WT, which was increased in each genotype by *SAUR15*-OE (Supplemental Figure S5C). These results were in contrast to what would be expected if SAUR15–BRI1 were prompting PM H^+^-ATPase activity by acting on the canonical BIN2-mediated pathway.

To further examine how SAUR15–BRI1 activates PM H^+^-ATPase, we tested for a physical interaction between BRI1 and PM H^+^-ATPase. An mbSUS two-hybrid assay indicated that BRI1 interacts strongly with Autoinhibited H^+^-ATPase isoform 1 (AHA1) and Autoinhibited H^+^-ATPase isoform 2 (AHA2) in yeast (Supplemental Figure S5D). BiFC experiments showed the location of this interaction was on the PM (Supplemental Figure S5E). co-IP assays using FLAG-tagged BRI1 transgenic seedlings indicated that BRI1 interacts with AHA in vivo, but there was neither increase nor decrease in the amount of co-immunoprecipitated AHA protein in BL- or BRZ-treated seedlings (Supplemental Figure S5, F and G). A similar amount of AHA protein was co-immunoprecipitated in Col-0, *saur15-1*, and *SAUR15-3HA* lines treated with or without BL or BRZ (Figure 5, D and E). However, immunoblotting showed that phosphorylation of penultimate residues in PM H^+^-ATPase was decreased in *bri1-301* and *bri1-701* mutants compared to wild-type Col-0 plants (Figure 5F; Supplemental Figure S5, H and I). In *SAUR15*-OE lines, phosphorylation of penultimate residues in PM H^+^-ATPase was partially restored in *bri1-301* but not in *bri1-701* mutants (Figure 5F; Supplemental Figure S5, H and I). Since BR promotes the phosphorylation of these penultimate residues in PM H^+^-ATPase (Minami et al., 2019), we propose that BR-induced SAUR15–BRI1 interaction leads to the direct phosphorylation and activation of H^+^-ATPases, thereby promoting cell expansion in plant organs.

## Discussion

**Figure 6:**
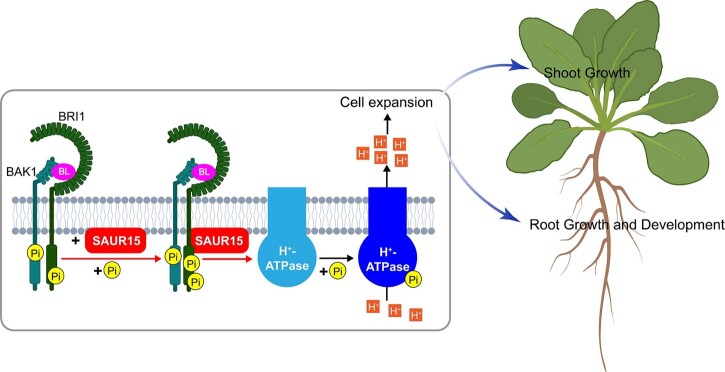
A hypothetical model for SAUR15–BRI1 mediated cell expansion in BR-regulated plant growth and development. The recruitment of SAUR15 by BR-activated BRI1 increases the phosphorylation of BRI1, which enhances the kinase activity of BRI1. Subsequently, BRI1 phosphorylates and activates PM H^+^-ATPases which promote cell expansion for plant growth and development.

BRs are essential for nearly all aspects of plant growth and development including cell expansion (Lv and Li, 2020; Nolan et al., 2020). At least part of this response is suggested to involve a rapid, BR signaling mechanism (Caesar et al., 2011). In this study, we identified SAUR15 as a key component of this response. Our data show that SAUR15–BRI1 interact in BR-treated plants to phosphorylate and activate PM-H^+^-ATPases leading to cell expansion (Figures 2, 5, and 6). Based on a combination of genetic, molecular, and biochemical data, we propose a working model for how SAUR15–BRI1 accomplish this task (Figure 6). Upon the perception of BR, the interaction of BRI1 and BAK1 triggers their phosphorylation (Wang et al., 2005, 2008), resulting in recruitment of SAUR15 (Figures 2, D and 6). This recruitment increases the phosphorylation and activation of BRI1 (Figures 2, E and F and 6). Subsequently, BRI1 activates PM H^+^-ATPases via phosphorylation to promote cell expansion-mediated plant growth and development (Figures 5 and 6; Supplemental Figure S5).

### The SAUR15–BRI1 module mediated a fast BR signaling pathway

SAURs have been implicated in various biological processes, including hypocotyl elongation, apical hook formation, and root growth and development (Spartz et al., 2014; Ren et al., 2018; Dong et al., 2019; Wang et al., 2020; Yin et al., 2020). All of these processes can be well-explained by SAUR-PP2C-D-PM H^+^-ATPase as a module for cell expansion (Takahashi et al., 2012; Spartz et al., 2014; Ren et al., 2018; Uchida et al., 2018; Minami et al., 2019; Yin et al., 2020). However, this SAUR-activated module is a downstream response of hormone and light signaling pathways. In brief, the perception of signals, such as auxin, BRs, or light, cause the expression of specific *SAUR* genes, generating accumulated SAUR proteins that form complex with PP2C-Ds to activate PM H^+^-ATPases (Sun et al., 2016; Dong et al., 2019; Minami et al., 2019; Wang et al., 2020). Strikingly, we show that SAUR15 functions at the top of the BR signaling pathway (Figure 6). Upon perception of BR, SAUR15 binds with BRI1 and promotes its activity (Figure 2). Activated BRI1 phosphorylates PM H^+^-ATPases to cause BR-mediated cell expansion required for growth (Figure 5). This fast response does not require BR-responsive gene expression and is completed entirely within the PM (Figure 6). Thus, we propose that SAUR15 integrates BR signaling responses via PM-H^+^-ATPase to promote rapid BR-mediated cell expansion, in addition to the classical BIN2-mediated signaling, which corroborates the theory proposed in past studies (Cerana et al., 1983, 1985; Caesar et al., 2011). It is possible that SAUR15-activated BRI1 also inhibits BIN2 to promote the expression of BR-responsive genes. How SAUR15–BRI1 selectively activates the two separate downstream pathways requires further study.

### SAUR15 is essential for SAUR15–BRI1-mediated BR signaling

Because of the SAUR15–BRI1 module, it is no surprise that the lateral root development phenotype of the *bin2-1*(−/−) mutant is opposite to that of *bri1-701* mutant (Cho et al., 2014; Figure 3, C and E; Supplemental Figures S2, D and F and S5, A and C). The absence of BRI1 in the *bri1-701* mutant blocks both BIN2 and SAUR15 modes of signaling. In contrast, SAUR15–BRI1–PM H^+^-ATPase signaling is preserved in the *bin2-1* (−/−) mutant as well as the positive regulation of continuously activated BIN2 on ARF7/ARF19 auxin response factors promoting lateral root formation (Cho et al., 2014; Yin et al., 2020). Our results also provide a reasonable explanation for earlier reports that BRI1–BIN2 plays a minor role in the regulating BIN2 activity during lateral root development (Figure 3, C and E; Supplemental Figure S5C), yet nearly all BRI1-related mutants show obvious lateral root developmental defects (Cho et al., 2014) (Supplemental Figure S2, D and F).

On the other hand, the rosette diameter of *bin2-1* (+/−) and *bri1-301*, as well as *bin2-1* (−/−) and *bri1-701* mutants is equally impaired (Supplemental Figure S4, A–C). This difference corresponds with lower expression of *SAUR15* and its homologs (Yin et al., 2020) in *bin2-1* shoots (Figure 4, C and E), expected to cause a dramatic decrease in BR-triggered BRI1 activation in shoots of *bin2-1* seedlings. Accordingly, *bin2-1* (−/−) defects can be substantially rescued by *SAUR15-*OE but not *BRI1-*OE (Figures 3, A and B and 4, A and B). These results demonstrate that SAUR15 and its homologs are likely essential for BRI1 phosphorylation and activation.

While SAUR15 promotes BR-induced plant growth, *SAUR15*-OE failed to rescue *bri1-701* and *cpd* defects (Figure 1; Supplemental Figure S2, D–S), suggesting that BR-triggered BRI1 is required for SAUR15 function. In accord, auxin was shown to have no effect on the activation and phosphorylation of PM H^+^-ATPase penultimate residues in BRZ-pretreated wild-type seedlings and *bri1-6* (Minami et al., 2019). This finding suggests that BR-triggered BRI1 is essential for auxin-induced SAUR15 activities and the SAUR–PP2C-D–PM H^+^-ATPase signaling module is no exception. Therefore, as downstream response genes of hormone and light signaling pathways, SAUR15 and its homologs, as well as the negative regulators of this family, are likely to exert function via SAUR–BRI1-mediated BR signaling pathway (Oh et al., 2014; Sun et al., 2016). Further experiments will decode the specific role of SAUR15 in activating BRI1 and how SAUR15 and BRI1 regulate each other.

In conclusion, BRs regulate a broad range of processes in plant growth and development and response to various stresses (Planas-Riverola et al., 2019; Nolan et al., 2020). In this study, we show that BR stimulates SAUR15–BRI1 interaction and signaling to promote cell expansion in roots and shoots via the phosphorylation and activation of PM H^+^-ATPases. Future studies will examine roles for SAUR15–BRI1 signaling in diverse tissues as well as in response to stresses. Our data reveal an alternate mode of BR signaling that drives cellular growth in plants.

## Materials and methods

### Plant materials and growth conditions

Arabidopsis (*Arabidopsis thaliana*) transfer DNA (T-DNA) insertion mutants *bri1-701* (SALK_003371), *cpd* (SALK_023532), and *cpd91* (SALK_078291) were obtained from the Arabidopsis Biological Resource Center. T-DNA insertion mutant *saur15-1* (GK-931C06) was obtained from GABI Kat (Yin et al., 2020). T-DNA mutants were PCR-genotyped as recommended. Point mutants *bin2-1* were genotyped using a dCAPS method (Li et al., 2001; Nam and Li, 2002). Primers are listed in Supplemental Table S1. Except for *bin2-*related seedlings, Col-0 was the background used as control. For *bin2*-related seedlings, WT seedlings were segregated from *bin2-1* (+/−) and used as control.

Plants were grown under long-day conditions (16-h white light per day, ∼100 μmol m^−2^ s^−1^ light intensity; 22°C ± 2°C) except for dark treatment. For root growth analysis, seeds were placed on 1/2 Murashige and Skoog (MS) medium containing 1% (w/v) sucrose with different concentrations of BL (E1641; Sigma, St. Louis, MO, USA) under long-day conditions. For hypocotyl growth analysis, seeds were grown on 1/2 MS medium containing 1% (w/v) sucrose with different concentrations of BRZ (SML1406; Sigma) under dark. For lateral root growth analysis, seedlings were grown on 1/2 MS medium containing 1% (w/v) sucrose under long-day conditions. For adventitious root growth analysis, 4-day-old dark-grown seedlings were transferred to long-day conditions. Medium acidification assays were performed as described previously (Yin et al., 2020). For liquid culture, seedlings were grown in flasks containing 50 mL of 1/2 MS medium containing 1% (w/v) sucrose. Photographs were taken with a digital camera. Root and hypocotyl lengths were measured using ImageJ software. Lateral and adventitious roots were counted with an Olympus light microscope (Yin et al., 2020).

### Generation of transgenic plants

Coding sequences of *SAUR15*, *BRI1*, and *BAK1* were polymerase chain reaction (PCR)-amplified and introduced into a Gateway *pDONR/ZEO* vector using BP clonase (Invitrogen, Waltham, MA, USA) then transferred into destination vectors with a 35S promoter and FLAG (*pBIB-35S-GWR-FLAG*), 3HA (*pBIB-35S-GWR-3HA*), or GFP (*pBIB-35S-GWR-GFP*) as epitope tags by LR clonase (Invitrogen) for plant expression. wild-type Col-0 plants were transformed by floral dipping as previously described (Yin et al., 2020). *35S::SAUR15-FLAG* was introduced into BR-related mutants for complementation experiments. *35S::SAUR15-GFP*, *35S::SAUR15-3HA*, *35S::BAK1-GFP*, and *35S::BRI1-FLAG* were used to create single or double transgenic plants. Transgenes were introgressed into mutant backgrounds by crossing. Homozygous plants were identified among F2 populations. Primers for generation and identification of transgenic plants are listed in Supplemental Table S1.

### RNA extraction and RT-qPCR

Shoot, root, and whole plant of 7-day-old seedlings or shoots of 14-day-old seedlings were collected and flash-frozen in liquid nitrogen. Total RNA was isolated by using an RNAprep Pure Plant Plus Kit (TIANGEN, Beijing, China) and cDNA was synthesized using a PrimeScript RT reagent Kit with gDNA Eraser (TaKaRa Biotechnology, Shiga, Japan). RT-qPCR was performed in triplicate or in duplicate on three bio-replicates using TB Green *Premix Ex Taq* II (Tli RNaseH Plus) (TaKaRa Biotechnology) on a StepOne Real-Time PCR Thermocycler (Applied Biosystems, Waltham, MA, USA). Primer-BLAST (www.ncbi.nlm.nih.gov/tools/primer-blast) was used for primer design and primer efficiency was verified by melt curve analysis. The transcript level of *SAUR19* subfamily genes (*SAUR19-24*) was analyzed by using homologous sequences in these genes for primer design. *ACTIN 2* (*ACT2*) was used as the internal control gene. Primers are listed in Supplemental Table S1.

### BiFC

The full-length (FL) coding sequences of *SAUR15*, *AHA1*, and *AHA2* were cloned into *pEarley Gate201*-*cYFP* (to create *SAUR15-YC*, *AHA1-YC*, and *AHA2-YC*), while FL *BRI1* was cloned into *pEarley Gate201*-*nYFP* (to create *BRI1-YN*). *Agrobacterium tumefaciens* strain *GV3101* harboring each plasmid was grown in Luria–Bertani broth containing 10-mM MES (pH 5.7) and 20-mM acetosyringone at 28°C. After shaking overnight, cells were collected and adjusted to an optical density at 600 nm (OD_600_) of 0.4 with MS liquid media containing 10-mM MES (pH 5.7), 10-mM MgCl_2_, and 150-mM acetosyringone. For co-infection, equal volumes of the appropriate resuspended cultures were mixed and incubated at 28°C for 2 h before injection into leaves of *N.benthamiana*. Samples were observed with a confocal laser scanning microscope (Leica TCS SP8) at 48 h after infiltration.  Yellow fluorescent protein (YFP) was excited at 488 nm and signals at 514–552 nm were collected. Laser intensity (8%) and detection settings (800 V Smart Gain) were kept constant.

### Yeast two-hybrid analysis

A mbSUS yeast (*Saccharomyces cerevisiae*) two-hybrid system was used to detect interactions between SAUR15, AHA1, and AHA2 with FL and truncated BRI1 according to the manual (Obrdlik et al., 2004). The FL coding sequences of *SAUR15*, *BAK1*, *AHA1*, and *AHA2* were mixed with linearized *pMetYCgate* vector and transformed into the haploid THY.AP4 yeast (SAUR15-Cub, BAK1-Cub, AHA1-Cub, and AHA2-Cub), while the FL coding sequences and truncated coding sequences, including ED and CD, of *BRI1* were mixed with linearized *pX-NubWTgate* vector and transformed into the haploid THY.AP5 yeast of the opposite mating type (BRI1-FL-Nub, BRI1-ED-Nub, and BRI1-CD-Nub). Diploid yeast cells were selected on synthetic complete (SC) medium with adenine and histidine (Ade + His). Interactions were assessed on synthetic dropout (SD) media containing 0, 150, or 400-mM methionine (Met). BAK1-Cub with BRI1-FL-Nub was used as a positive control (Li et al., 2002) and empty vector pair *pMetYCgate* with *pXNubWTgate* was used as a negative control (Obrdlik et al., 2004). Primers for cloning are listed in Supplemental Table S1.

### co-IP

To analyze interaction between BRI1 with SAUR15, BAK1, and AHA under BL treatment, 10-day-old seedlings treated with or without 1-μM BL for 2 h were ground to a fine powder in liquid nitrogen. Membrane proteins were extracted as previously described (Li et al., 2002). BRI1-FLAG were immunoprecipitated from the solubilized membrane fraction with α-FLAG M_2_ affinity gel (A2220; Sigma). Immunoprecipitated proteins were separated by 8% (w/v) or 10% (w/v) bis-(2-hydroxyethyl)amino-tris(hydroxymethyl)methane polyacrylamide gel electrophoresis (Bis–Tris PAGE) for Western blot analysis with α-GFP (11814460001; Roche, Basel, Switzerland), α-FLAG (M20008; Abmart Shanghai, China), and α-AHA (AS07; Agrisera Vännäs, Sweden). To analyze interaction between BRI1 and AHA under BRZ treatment, 9-day-old seedlings treated with or without 1-μM BRZ for 18 h were ground to a fine powder in liquid nitrogen. Membrane proteins were extracted as previously described (Li et al., 2002). BRI1-FLAG were immunoprecipitated from the solubilized membrane fraction with α-FLAG M_2_ affinity gel. Immunoprecipitated proteins were separated by 10% (w/v) Bis–Tris PAGE for Western blot analysis with α-AHA antibodies.

### Phosphorylation assays

To analyze phosphorylated BRI1 in vivo, 10-day-old 1/2 MS liquid-cultured seedlings treated with or without 100 nM BL for 90 min were ground to a fine powder in liquid nitrogen. The membrane protein was extracted as previously described (Li et al., 2002). BRI1-FLAG protein was immunoprecipitated from the solubilized membrane fraction with α-FLAG M_2_ affinity gel. Immunoprecipitated proteins were separated by 8% (w/v) Bis–Tris PAGE. Phosphorylated BRI1 was detected with an α-pThr antibody (9381; Cell Signaling Technology, Danvers, MA, USA). To analyze AHA protein, PM proteins extracted from 7-day-old seedlings were separated by 10% (w/v) Bis–Tris PAGE. AHA protein was detected by immunoblotting using α-AHA antibodies. The phosphorylation state of the penultimate residue of AHA was estimated using an α-pThr947 antibody (prepared by Abmart Biotechnology) following the methods of Takahashi et al. (2012). To analyze phosphorylation level of BZR1, 7-day-old seedlings grown on 1/2 MS medium treated with or without 1 μM BL for 120 min were ground to a fine powder in liquid nitrogen. The total protein was extracted as previously described (Gou et al., 2012) and the phosphorylated and unphosphorylated BZR1 was detected with an α-BZR1 antibody (YXZPK82; Youke, Beijing, China).

### Apoplastic pH measurement

Apoplastic pH was measured according to Cho et al. (2012). At least 50 rosette leaves per genotype were submerged in 50 mL of water and subjected to 4 cycles of 5-min vacuum followed by rapid release. Then, the leaves were dried with filter paper and put into a 5-mL syringe without plunger. The exhaust of the syringe linked to a 0.5-mL conical tube was placed into a 50-mL centrifuge tube. The whole mounting was centrifuged at 1,000 *g* for 10 min at 4°C. About 160 μL of apoplastic fluid was mixed with 40 mL 100-mg mL^−1^ 8-hydroxypyrene-1,3,6-trisulfonic acid trisodium (H1529; Sigma) and fluorescence was detected at 510 and 530 nm using an excitation wavelength of 460 nm. A standard curve was made with Britton–Robinson universal buffer containing 0.05 M H_3_BO_3_, 0.05 M H_3_PO_4_, 0.05 M CH_3_COOH, which was adjusted to pH ranging from 4.5 to 8.0 with NaOH.

### ATPase activity assays

PM proteins were extracted from 7-day-old seedlings grown on 1/2 MS medium containing 1% (w/v) sucrose according to Minami et al. (2017). Protein concentration was determined using the method of Bradford (1976). PM H^+^-ATPase activity was analyzed by the method of Xu et al. (2012) using 0.5 mL of reaction solution [3 mg of PM protein in 0.02% (w/v) Brij 58, 30 mM 1,3-bis(tris(hydroxymethyl)methylamino)-propane/MES pH 6.5, 5 mM MgSO_4_, 50-mM KCl and 4 mM Tris-ATP] per assay. Reactions went for 30 min at 30°C after which 1 mL of stopping solution containing 2% (v/v) concentrated H_2_SO_4_, 5% (w/v) SDS, 0.7% (w/v) sodium molybdate, and 50 mL of 10% (w/v) ascorbic acid was added. After 30 min, color development of the phosphomolybdate complex was measured at OD_700_ with a spectrophotometer. PM H^+^-ATPase activity was calculated as the phosphorus liberated in excess of boiled-membrane controls. To assess the purification of the PM, the result was expressed as the activity difference with or without 0.1-mM sodium vanadate. The experiment was repeated 3 times and each biological replicate includes ˃10 g seedlings.

### Statistical analysis

Data are reported as the mean ± standard deviation. Statistical analysis was performed using one-way analysis of variance with Tukey’s test as implemented in IBM SPSS Statistics version 22.0 software.

### Accession numbers

Sequence data of genes in this study can be found in the EMBL/Genbank data libraries under accession numbers *SAUR15* (AT4G38850), *BRI1* (AT4g39400), *BAK1* (AT4g33430), *BIN2* (AT4G18710), *CPD* (AT5G05690), *DWF4* (AT3G50660), *BZR1* (AT1G75080), *SAUR19* (AT5G18010), *SAUR20* (AT5G18020), *SAUR21* (AT5G18030), *SAUR22* (AT5G18050), *SAUR23* (AT5G18060), *SAUR24* (AT5G18080), *AHA1* (AT2G18960), *AHA2* (AT4G30190), and *ACT2* (AT3G18780).

## Supplemental data 

The following materials are available in the online version of this article.

**Supplemental Figure S1.** SAUR15 is a positive regulator of BR signaling responses.

**Supplemental Figure S2.** *SAUR15*-OE can partially suppress the phenotype of BR signaling and biosynthesis mutants.

**Supplemental Figure S3.** SAUR15 could function in a separate pathway from BIN2.

**Supplemental Figure S4.** Phenotype comparison of *bin2*, *bri1*, and *cpd* mutants.

**Supplemental Figure S5.** BRI1 interacts with PM H^+^-ATPases and regulates their activity.

**Supplemental Table S1.** Primers used in this study.

## Supplementary Material

kiac194_Supplementary_Materials

## Notes

H.Y. and S.M.W. conceived and guided the study. H.Y. and M.L. designed the experiments. M.L., H.Y., C.L., C.M., and H.L. performed the experiments. H.Y. and M.L. analyzed the data and interpreted the results. H.Y. and M.L. wrote the manuscript. S.R.H. and J.L. read and edited the manuscript.

The author responsible for distribution of materials integral to the findings presented in this article in accordance with the policy described in the Instructions for Authors (https://academic.oup.com/plphys/pages/general-instructions) is: Hongju Yin (yinhj@lzu.edu.cn).
