# Supplementary material for: SAUR15 interaction with BRI1 activates plasma membrane H+-ATPase to promote organ development of Arabidopsis
Source: Plant Physiol. 2022 May 2;189(4):2454–66. doi: 10.1093/plphys/kiac194 (PMC9343009; doi:10.1093/plphys/kiac194)
Supplement: kiac194_Supplementary_Materials [file kiac194_supplementary_materials.pdf]

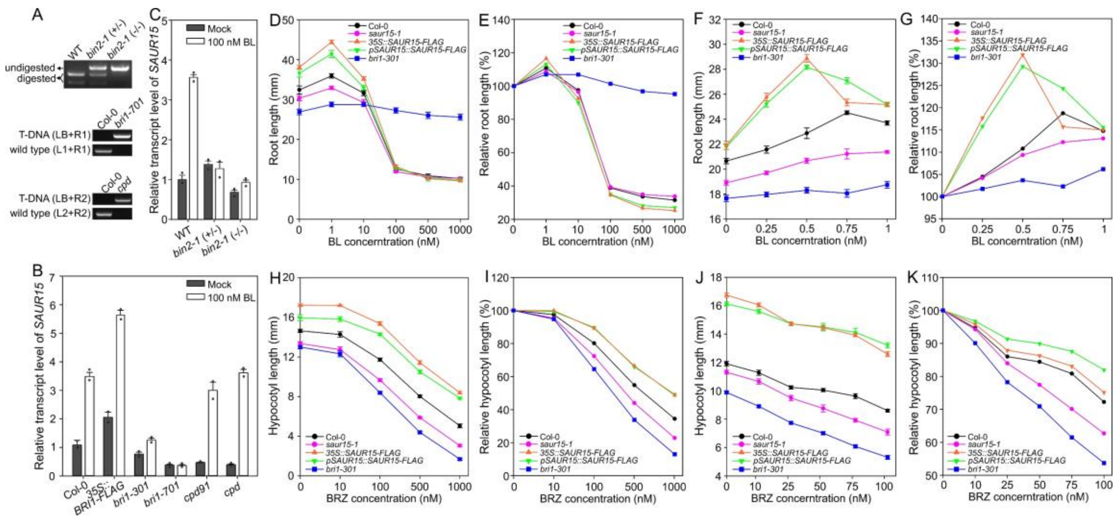

Supplemental Figure S1. SAUR15 is a positive regulator of BR signaling responses. A, Derived cleaved amplified polymorphic sequence (dCAPS) analysis of *bin2-1* and genotyping of *bri1-701, cpd*. B and C, Relative transcript level of *SAUR15* in BR signaling and biosynthesis related seedlings with or without 100 nM 2,4-epibrassinolide (BL) treatment for 90 min. *ACT2* was used as the reference gene. Primers used in (A-C) are listed in Supplemental Table S1. D and F, Root growth analysis of Col-0, *saur15-1*, *SAUR15*-OE (*35S::SAUR15-FLAG* and *pSAUR15::SAUR15-FLAG*), and *bri1-301* seedlings grown on ½ MS medium containing different BL concentrations. E and G, Relative root length of seedlings comparing to that of 0 nM BL treatment in (D, F). H and J, Hypocotyl growth analysis of Col-0, *saur15-1*, *SAUR15*-OE*,* and *bri1-301* seedlings grown in dark on ½ MS medium containing different brassinazole (BRZ) concentrations. I and K, Relative hypocotyl length of seedlings comparing to that of 0 nM BRZ treatment in (H, J). For (B, C), data shown are mean ± SD of two technical replicates. Experiments were repeated three times with similar results. Each biological replicates include 10 seedlings. For (D, F, H, J), data are mean ± SD (n = 10).

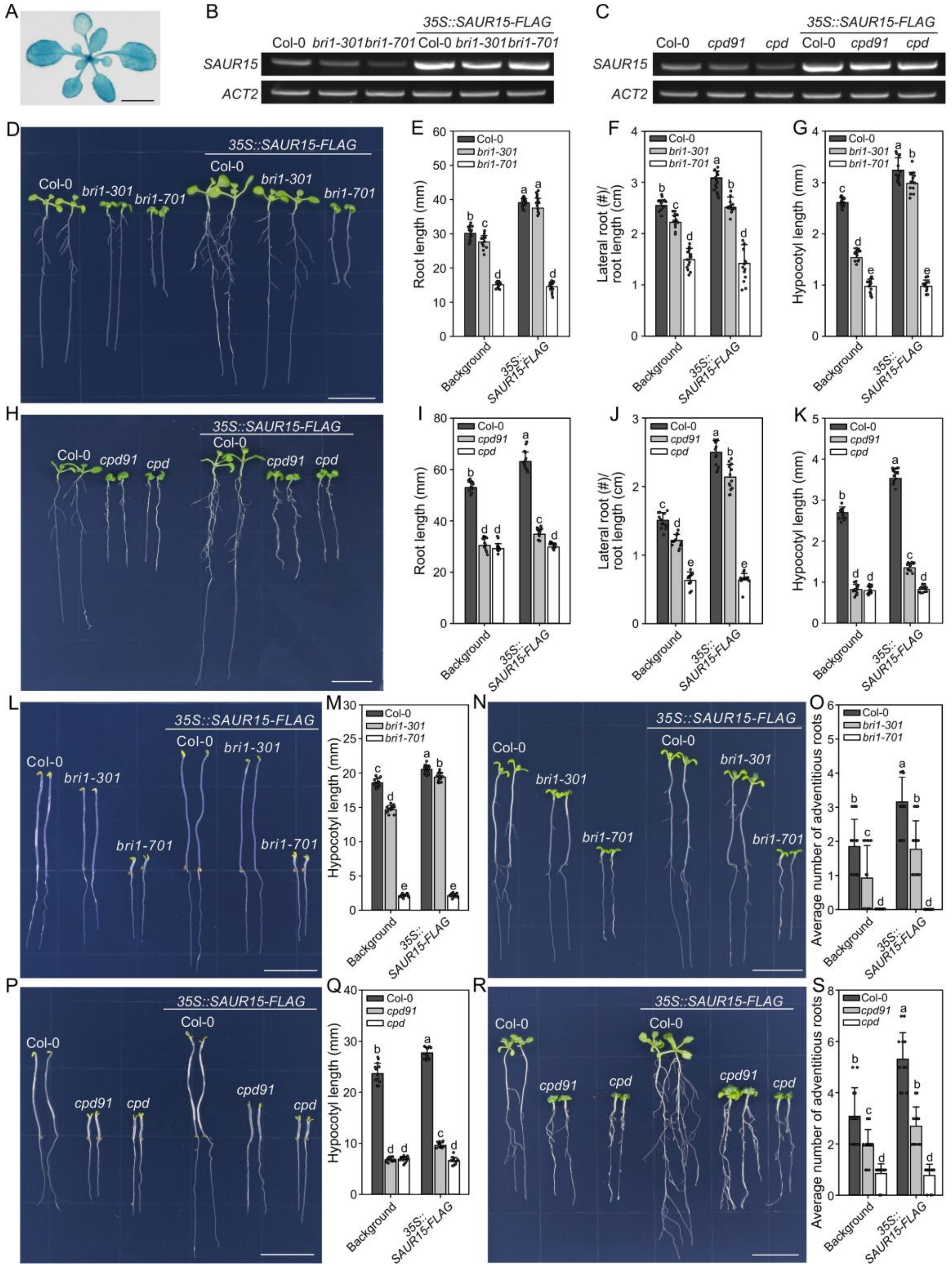

Supplemental Figure S2. *SAUR15*-OE can partially suppress the phenotype of BR signaling and biosynthesis mutants. A, GUS staining of 3-week-old *pSAUR15::GUS* transgenic seedlings. B, Expression level of *SAUR15* in Col-0, *bri1-301*, *bri1-701* and *SAUR15*-OE lines grown on ½ MS medium. C, Expression level of *SAUR15* in Col-0, *cpd91*, *cpd* and *SAUR15*-OE lines grown on ½ MS medium. For (B, C), *ACT2* was used as the reference gene. Primers used are listed in Supplemental Table S1. D and H,

Phenotypes of 10-day-old seedlings grown on ½ MS medium in light. E-G and I-K, Primary root length (E, I), lateral root density (F, J) and hypocotyl length (G, K) of seedlings shown in (D, H). L and P, Phenotypes of seedlings grown on ½ MS medium in dark for 4 days. M and Q, Hypocotyl growth analysis of seedlings shown in (L, P). N and R, Adventitious root phenotypes. 4-day-old dark-grown plants were moved into light and grown for another 7 (N) or 10 (R) days. O and S, Statistical analysis of adventitious roots of plants in (N, R). Data shown in (E-G, I-K, M, O, Q, S) are mean ± SD (n = 13). Different letters indicate significant differences (one-way ANOVA with Tukey's test, $P < 0.05$). Scale bars, 1 cm.

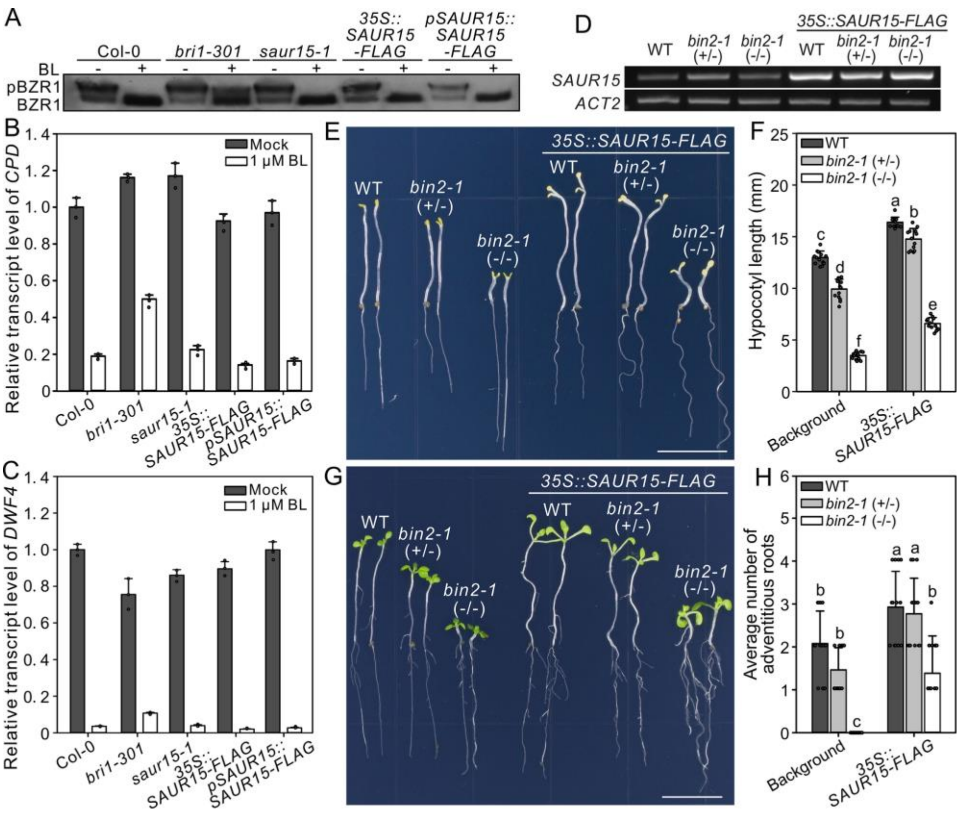

Supplemental Figure S3. SAUR15 could function in a separate pathway from BIN2. A, Phosphorylation state of BZR1 in 7-day-old seedlings of Col-0, *bri1-301*, *saur15-1* and *SAUR15*-OE lines treated with or without 1 μM 2,4-epibrassinolide (BL) for 120 min. B and C, Relative transcript level of *CPD* (B) and *DWF4* (C) in 7-day-old seedlings of Col-0, *bri1-301*, *saur15-1* and *SAUR15*-OE lines treated with or without 1 μM BL for 120 min. D, Expression level of *SAUR15* in wild type (WT), *bin2-1* (+/-), *bin2-1* (-/-) and *SAUR15*-OE lines grown on ½ MS medium. For (B-D), *ACT2* was used as the reference gene. Primers are listed in Supplemental Table S1. E, Phenotypes of seedlings grown on ½ MS medium in dark for 4 days. F, Hypocotyl growth analysis of seedlings described in (E). G, Adventitious root phenotypes. 4-day-old dark-grown plants were transferred to light and grown for another 7 days. H, Statistical analysis of adventitious root of plants in (G). For (B, C), data show mean ± SD of three technical replicates. Experiments were repeated three times with similar results. Each biological replicates include 10 seedlings. For (F, H), data are mean ± SD (n = 13). Different letters indicate significant differences (one-way ANOVA with Tukey's test, $P < 0.05$). Scale bars, 1 cm.

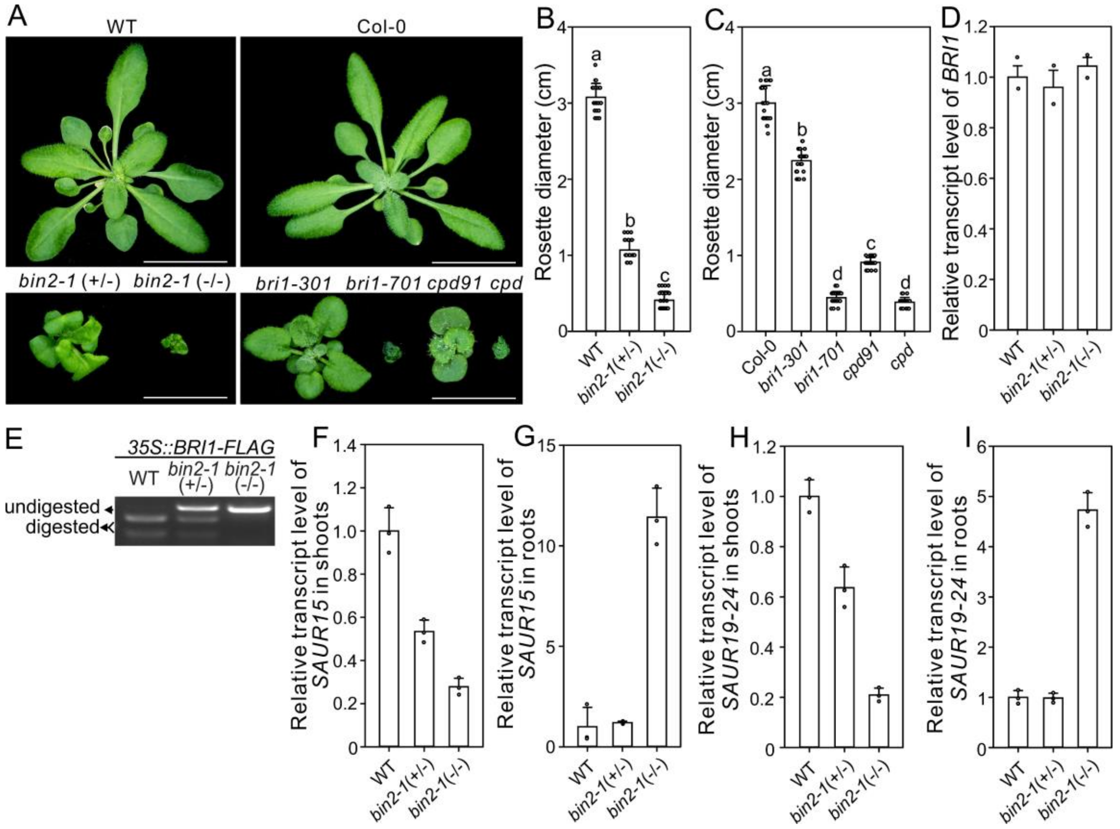

Supplemental Figure S4. Phenotype comparison of *bin2*, *bri1*, and *cpd* mutants. A, Rosette leaves of wild-type and mutants grown in soil for 3 weeks. Scale bars, 1 cm. B and C, Rosette diameter of seedlings in (A). D, Relative transcript level of *BRI1* in shoots of wild-type, *bin2-1* (+/-) and *bin2-1* (-/-) seedlings grown in soil for 2 weeks. E, dCAPS analysis confirming *bin2-1* status of *BRI1*-OE (*35S::BRI1-FLAG*) lines. F and G, Relative transcript level of *SAUR15* in shoots (F) and roots (G) of *bin2* seedlings grown on ½ MS medium for 1 week. H and I, Relative transcript level of *SAUR19-24* in shoots (H) and roots (I) of *bin2* seedlings grown on ½ MS medium for 1 week. For (D, F-I), *ACT2* was used as the reference gene. Primers used in (D-I) are listed in Supplemental Table S1. In (B, C), data are mean ± SD (n = 25). In (D), data are mean ± SD of two technical replicates. In (F-I), data are mean ± SD of three technical replicates. Experiments were repeated three times with similar results. For (F,H), Each biological replicates include shoots from 10 seedlings. For (G,I), Each biological replicates include roots from 30 seedlings. Different letters indicate significant differences (one-way ANOVA with Tukey's test, $P < 0.05$).

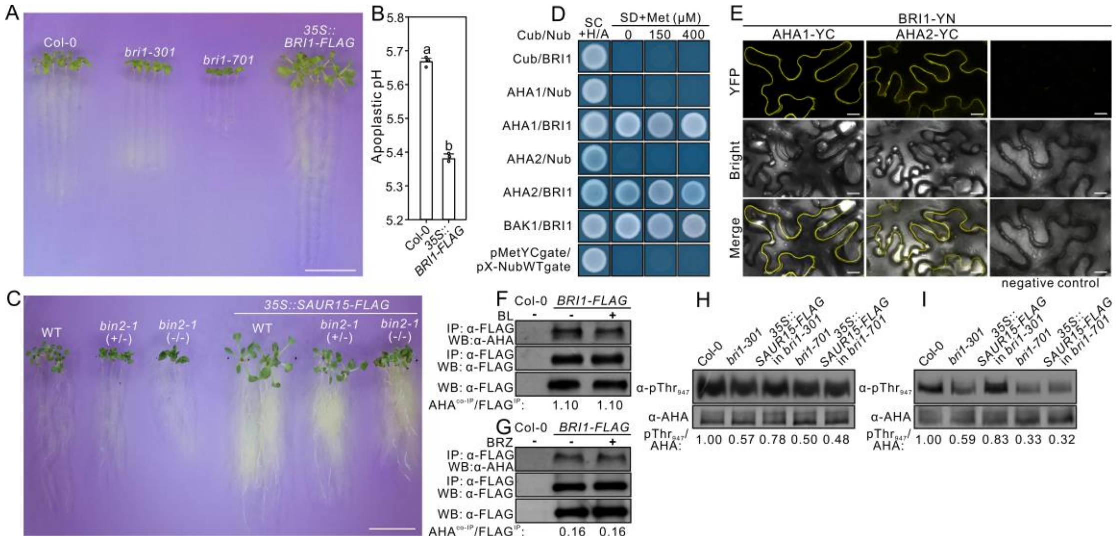

Supplemental Figure S5. BRI1 interacts with PM H^+-ATPases and regulates their activity. A, Medium acidification assays of BRI1 related seedlings. 12-day-old seedlings were transferred to plates containing the pH indicator dye bromocresol purple. A change in color from purple to yellow indicates a decline in pH. Color changes were recorded after 24 h. Scale bars, 1 cm. B, Leaf apoplastic pH. Absorbance of 8-hydroxypyrene-1,3,6-trisulfonic acid trisodium was measured at 510 and 530 nm. At least 50 leaves were used for each replicate. C, Medium acidification assays of *bin2-1* and *SAUR15*-OE seedlings. Medium acidification was analyzed as described in (A). Scale bars, 1 cm. D, Interaction assay between BRI1 and PM H^+-ATPases AHA1 and AHA2 using a yeast split-ubiquitin system (mbSUS). BRI1 proteins were fused to the N-terminal part of ubiquitin (Nub). AHA1, AHA2, and BAK1 proteins were fused to the C-terminal part of ubiquitin (Cub). Yeast was grown on a synthetic complete medium containing Ade and His (SC + Ade + His) for selection of diploid cells or on a synthetic dextrose minimal medium (SD) with 150 and 400 mM or without Met for interaction detection. BRI1-Nub with BAK1-Cub was used as a positive control and empty vector pair pMetYCgate with pX-NubWTgate was used as a negative control. E, BRI1 interacts with AHA1 and AHA2 in *Nicotiana benthamiana* leaf epidermal cells. BRI1 was fused to the N-terminal part of YFP (YN); AHA1 and AHA2 were fused to the C-terminal part of YFP (YC), respectively. *GV3101* harboring BRI1-YN was used as the negative control. Scale

bars, 10 μm. F, *In vivo* co-immunoprecipitation of BRI1-FLAG with AHA in transgenic plants with or without 2,4-epibrassinolide (BL) treatment. 10-day-old liquid-cultured seedlings were collected and treated with 1 μM BL for 2 h. Total membrane protein was immunoprecipitated and subjected to immunoblot analysis as indicated. G, *In vivo* co-immunoprecipitation of BRI1-FLAG with AHA in transgenic plants treated with or without brassinazole (BRZ). 9-day-old liquid-cultured seedlings were collected and treated with 1 μM BRZ for 18 h. Total membrane protein was immunoprecipitated and subjected to immunoblot analysis as indicated. H and I, Replicate experiments for AHA phosphorylation level analysis in Fig. 5F. The value of $pThr_{947}$/AHA in Col-0 were set to 1.00 and values for other lines were normalized. For (B), data show mean ± SD of three technical replicates. Different letters indicate significant differences (one-way ANOVA with Tukey's test, $P < 0.05$). For (F, G), IP, immunoprecipitation, WB, western blot. For (F-I), the $AHA^{co\text{-}IP}/FLAG^{IP}$ ratio in (F, G) and the $pThr_{947}$/AHA ratio (H, I) were measured by western blot analysis using ImageJ.

Supplemental Table S1. Primers used in current study. The underlined sequences represent the recombination sites.

**Primers**	**Sequences**
**For genotyping of T-DNA lines**	
R1	GATTCCTTTCCTCGGAGATTG
L1	TTGTTCGGATCTGATTCCTTG
R2	CTCCTCCCTCCTTTTCATCAC
L2	GAAAAGAAGAAGAATGCAAAAGAG
LBb1.3	ATTTTGCCGATTTCGGAAC
**For genotyping of point mutants**	
bin2-1-dCAPS-F	CCGGAGAAAATGCTGTGGATC
bin2-1-dCAPS-R	GCCAGGGATGTGCCTTTATCTG
**For RT-qPCR and semi-quantitative RT-PCR**	
SAUR15-QF	TGTTCTTCACAGTCACATCTCAG
SAUR15-QR	ATCTCCAGGGGAAATTAGCTAACA
SAUR15-semi-QF	GGCTTTTTTGAGGAGTTTC
SAUR15-semi-QR	TATCTGAGATGTGAC
CPD-QF	CAACCCTTGGAGATGGCAGA
CPD-QR	AACCCTAGCCAGCTCGTAAC
DWF4-QF	TCCCTAGTGGGTGGAAAGTG
DWF4-QR	GCTCCGTTGTTTTGCTGTTG
BRI1-QF	CTCTCCTGTCTCTCACCGGA
BRI1-QR	GCACTTGAAGCCAGAAACGG
SAUR19-24-QF	AGATTCTAAGCCGCTCCACC
SAUR19-24-QR	TCAAGTATGAGAGCGGCACC
ACT2-QF	TCAGATGCCCAGAAGTGTTGTTCC
ACT2-QR	CCGTACAGATCCTTCCTGATATCC
ACT2-semi-QF	TGGAAAAGATCTGGCATCAC
ACT2-semi-QR	TCAAGACGGAGGATGGCATG
**For coding sequence cloning**	
SAUR15-Full-F	AAAAAGCAGGCTTCATGGCTTTTTTGAGGAGTTTC
SAUR15-Full-R	AGAAAGCTGGGTTTTGTATCTGAGATGTGAC
AHA1-Full-F	AAAAAGCAGGCTTCATGTCAGGTCTCGAAGATAT
AHA1-Full-R	AGAAAGCTGGGTTCACAGTGTAGTGATGTCCTG
AHA2-Full-F	AAAAAGCAGGCTTCATGTCGAGTCTCGAAGATATC
AHA2-Full-R	AGAAAGCTGGGTTCACAGTGTAGTGACTGGGA
BRI1-Full-F	AAAAAGCAGGCTTCATGAAGACTTTTTCAAGCTTC
BRI1-Full-R	AGAAAGCTGGGTTTAATTTTCCTTCAGGAACTTC
BRI1-ED-F	AAAAAGCAGGCTTCATGAAGACTTTTTCAAGCTTC
BRI1-ED-R	AGAAAGCTGGGTTTCTACCAACAAGGATCAG
BRI1-CD-F	AAAAAGCAGGCTTCATGGAGATGAGGAAGAGAC
BRI1-CD-R	AGAAAGCTGGGTTTAATTTTCCTTCAGGAACTTC
BAK1-Full-F	AAAAAGCAGGCTTCATGGAACGAAGATTAATGATC

BAK1-Full-R	AGAAAGCTGGGTTTCTTGGACCCGAGGGGTATTC
attB1	ACAAGTTTGTACAAAAAAGCAGGCT
attB2	ACCACTTTGTACAAGAAAGCTGGGT
